# Alkaline twin-screw extrusion pretreatment for fermentable sugar production

**DOI:** 10.1186/1754-6834-6-97

**Published:** 2013-07-08

**Authors:** Chao Liu, Evert van der Heide, Haisong Wang, Bin Li, Guang Yu, Xindong Mu

**Affiliations:** 1 Key Laboratory of Biofuels, Key Laboratory of Biobased Materials, Qingdao Institute of Bioenergy and Bioprocess Technology, Chinese Academy of Sciences, Qingdao 266101, China; 2 Shell Global Solutions International B.V, Shell group, Carel van Bylandtlaan 30, Hague 2596 HR, Netherlands

**Keywords:** Twin-screw extrusion, Pretreatment, Corn stover, Sugar recovery, Enzymatic hydrolysis

## Abstract

**Background:**

The inevitable depletion of fossil fuels has resulted in an increasing worldwide interest in exploring alternative and sustainable energy sources. Lignocellulose, which is the most abundant biomass on earth, is widely regarded as a promising raw material to produce fuel ethanol. Pretreatment is an essential step to disrupt the recalcitrance of lignocellulosic matrix for enzymatic saccharification and bioethanol production. This paper established an ATSE (alkaline twin-screw extrusion pretreatment) process using a specially designed twin-screw extruder in the presence of alkaline solution to improve the enzymatic hydrolysis efficiency of corn stover for the production of fermentable sugars.

**Results:**

The ATSE pretreatment was conducted with a biomass/liquid ratio of 1/2 (w/w) at a temperature of 99°C without heating equipment. The results indicated that ATSE pretreatment is effective in improving the enzymatic digestibility of corn stover. Sodium hydroxide loading is more influential factor affecting both sugar yield and lignin degradation than heat preservation time. After ATSE pretreatment under the proper conditions (NaOH loading of 0.06 g/g biomass during ATSE and 1 hour heat preservation after extrusion), 71% lignin removal was achieved and the conversions of glucan and xylan in the pretreated biomass can reach to 83% and 89% respectively via subsequent enzymatic hydrolysis (cellulase loading of 20 FPU/g-biomass and substrate consistency of 2%). About 78% of the original polysaccharides were converted into fermentable sugars.

**Conclusions:**

With the physicochemical functions in extrusion, the ATSE method can effectively overcome the recalcitrance of lignocellulose for the production of fermentable sugars from corn stover. This process can be considered as a promising pretreatment method due to its relatively low temperature (99°C), high biomass/liquid ratio (1/2) and satisfied total sugar yield (78%), despite further study is needed for process optimization and cost reduction.

## Background

With the gradual short supply of petroleum source, it has been a hot research field in exploitation and utilization of lignocellulosic biomass such as the wastes of agriculture and forestry (e.g., corn stalk, rice straw, wheat straw, bagasse, saw dust, etc.) by converting them into liquid fuels or chemicals, as it is of great importance to establish a circular economy mode of sustainable development.

However, the enzymatic conversion of carbohydrates in lignocellulosic biomass to fermentable sugars is difficult as these sugar-based polymers are compactly associated with lignin [1,2]. Some structural factors, such as content of lignin, hemicelluloses, and acetyl group, cellulose crystallinity, degree of polymerization, accessible surface, etc., can impact enzymatic hydrolysis to different extent [3-6]. The crystallinity or degree of polymerization contributes to the recalcitrance of lignocellulosic biomass to hydrolysis, but they alone are insufficient to prevent significant hydrolysis [1]. Recently, hemicellulose removal was found to be more important than removal of lignin [7]. Anyhow, pretreatment is required to disrupt the natural recalcitrance of lignocellulosic biomass for effective enzymatic saccharification.

Many chemical, mechanical, thermo-chemical and biochemical pretreatment methods have been studied and are still in the development with varying levels of success, including acid hydrolysis, alkali hydrolysis, the organosolv process, steam explosion, ammonia fiber explosion (AFEX), hot water treatment, and microorganism treatment [8-11]. However, currently available pretreatment techniques can hardly meet the requirements of commercial application due to long processing times, chemical recycle problem, or high operational cost [9,12].

Extrusion pretreatment is a novel physical-chemical method in which biomass is processed by means of heat, compression and shear forces, leading to physical disruption and chemical modifications of biomass during the passage through the extruder. Various types of extrusion processes have been studied for the pretreatment of biomass and the extrusion pretreatment is considered as a promising technology for biomass conversion to ethanol production in recent studies [13]. Lee SH, Teramoto Y and Endo T [14] used a batch-type kneader with twin-screw elements in conjunction with hot water process for pretreatment of woody biomass. Karunanithy C and Muthukumarappan K [15] used a single screw extruder with different screw speeds and temperatures for pretreatment of corn stover. Kadam KL, Chin CY and Brown LW [16] developed a two stage twin-screw extrusion process for producing ethanol and low-molecular-weight lignin. However, there still are some handicaps in some cases, such as the low treatment rate, low biomass/liquid ratios or relatively high temperature [17]. In this study, a specially designed screw extruder was developed, and it consists of a series of transport screws and reversed screws, which are different from the ones mentioned above (The details of the screws were described in section of Methods). The current work is expected to establish an effective pretreatment method via using the specially designed extruder, which can be conducted at a relatively low temperature with high biomass/liquid ratio, to overcome the recalcitrance of lignocellulose.

**Figure 1:**
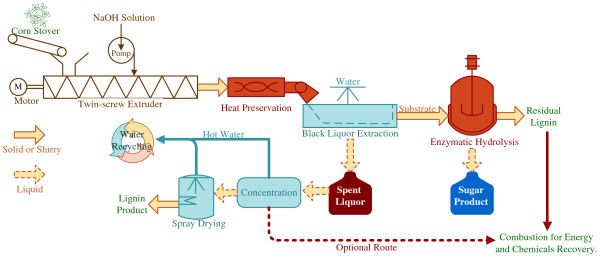
Schematic diagram of the overall ATSE pretreatment system.

The schematic diagram of the ATSE process is shown in Figure 1. The corn stover was pretreated by the specially designed extruder followed by heat preservation, washing and enzymatic hydrolysis, while the processes of concentration and water reuse were not performed in this study, and the enzymatic hydrolysis tests were conducted at the solid loading of 2% to estimate the effectiveness of ATSE pretreatment. The spent liquor can be easily recovered to produce alkali lignin or combusted to recover the chemicals and energy using existing industrial technology (i.e. black liquor evaporation) well developed in pulp mills [18]. In this research, the effects of key parameters (alkali charge and heat preservation after extrusion) of ATSE pretreatment on the composition changes and enzymatic digestibility of corn stover were investigated, based on the experimental data from pilot trial.

## Results and discussion

### Composition changes of corn stover after ATSE pretreatment

**Figure 2:**
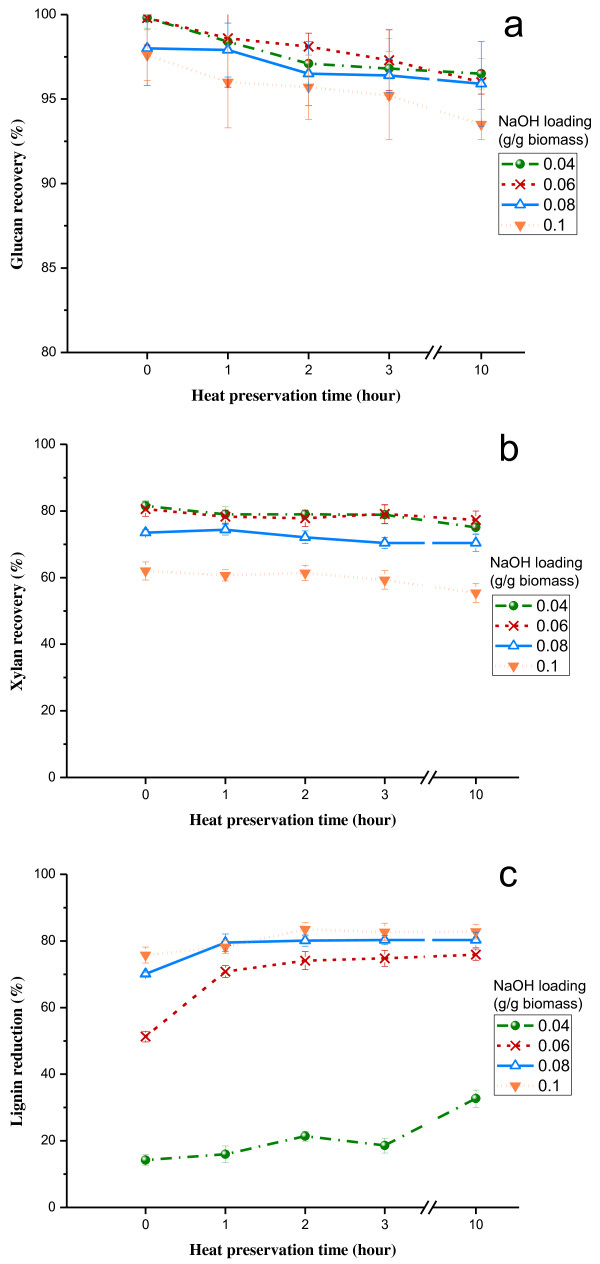
**Effects of ATSE pretreatment under different conditions on glucan recovery (a), xylan recovery (b) and delignification (c).** Recovery_glucan/xylan_ (%) = M_glucan/xylan of pretreated biomass_/M_glucan/xylan of original biomass_. Lignin reduction (%) = 1 – M_lignin of pretreated biomass_/M_lignin of original biomass_. M is mass of sugar/lignin.

The effects of ATSE pretreatment under different conditions on glucan, xylan and lignin content of the pretreated corn stover are shown in Figure 2, which presents that ATSE pretreatment can drastically change the composition of corn stover. Figure 2a shows that the ATSE pretreatment only caused minor glucan loss under the certain NaOH loading and heat preservation time, and about 96% of the glucan present in the untreated biomass was recovered after ATSE pretreatment. The good preservation of glucan might due to its semi crystalline structure [19].

Hemicellulose has amorphous, heterogeneous and branched structure with little strength, which makes it more susceptible to solubilization than cellulose at alkaline conditions [19]. The solubilization of xylan displayed an obvious difference from glucan degradation during ATSE pretreatment as shown in Figure 2b. Portion of xylan was degraded and removed during the pretreatment, and the removal increased with the increase of NaOH loading. For instance, ATSE treatment with NaOH loading of 0.06 g/g biomass (oven dried) and heat preservation time of 1 h resulted in 21.7% loss of the xylan, and the loss increased to 39.3% when NaOH loading increased to 0.1 g/g biomass. The xylan recovery tended to decrease with the increase of heat preservation time, but the impact of heat preservation time was limited. Carbohydrate loss into spent liquor would result in a waste of available sugar in solid residue and it should be avoided as much as possible. Therefore, the excessively high NaOH loading (over 0.1 g/g biomass) which cause lots of xylan degradation was not a good choice for overall sugar recovery.

Lignin is a three dimensional complex aromatic polymer which forms a sheath surrounding cellulose and hemicellulose. It acts as a physical barrier, restricts cellulase access to cellulose and, thereby, reduces the activity of the enzyme through non-productive binding [20]. Several studies have demonstrated strong positive correlations between delignification and sugar released from enzymatic hydrolysis [21]. The degree of delignification reflects the effectiveness of the alkaline pretreatment process and it is critical to improve enzymatic digestibility of lignocelluloses [11]. Unlike structural carbohydrate, the lignin content of the biomass was significantly reduced after ATSE pretreatments and the lignin reduction increased from 14% to 83% as the pretreatments were intensified (from 0.04 to 0.1 g NaOH/g biomass), as shown in Figure 2c, but the lignin removal increased slowly with extending heat preservation. For instance, only about 5% improvement of delignification could be obtained when extending heat preservation time from 1 to 10 h with the NaOH loading of 0.06 g/g biomass. In addition, relatively good lignin reduction of about 70% and 80% could be achieved at NaOH loading of 0.06 and 0.08 g/g biomass, respectively, with the heat preservation time of 1 h. However, ATSE treatment with NaOH loading of 0.04 g/g biomass didn’t work well in delignification. More importantly, Figure 2c also exhibits that, even without heat preservation, around 50% to 75% lignin reduction could be reached after ATSE pretreatment with NaOH loading of 0.06 to 0.1 g/g biomass. This was likely due to the increase of specific surface area and the fibrillation of the biomass under the physical and chemical reactions during the ATSE pretreatment [22], leading to the faster removal of lignin with the treatment of NaOH.

### Effect of ATSE pretreatment on the enzymatic digestibility of corn stover

**Figure 3:**
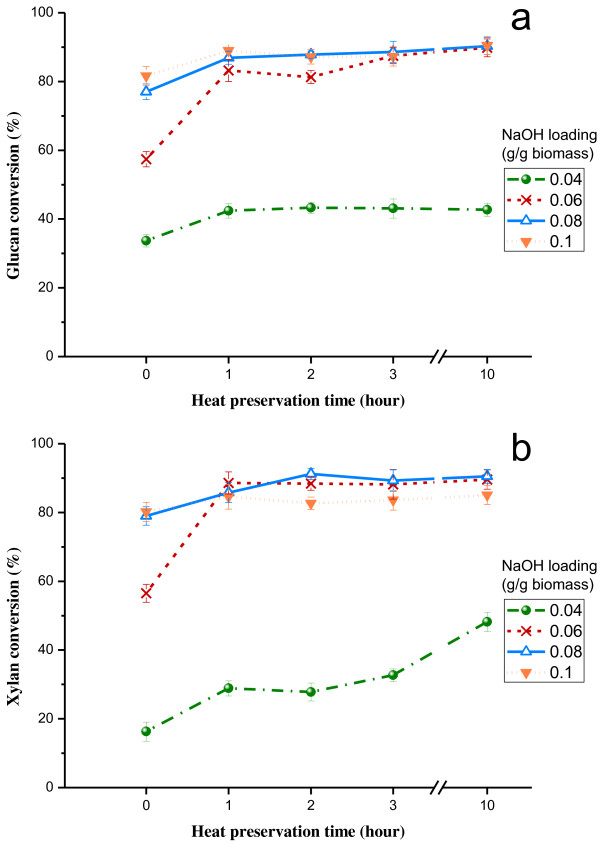
**Effect of ATSE pretreatment on glucan and xylan conversions.** The conversions are based on the sugar content in the pretreated material. Enzymatic hydrolysis conditions: 2% of solid concentration with 20 FPU/substrate of cellulase and 5 IU/substrate of beta-glucosidase; 50°C for 48 h.

Hydrolysis of both cellulose and hemicellulose in pretreated biomass via enzymatic action is critical to release monomeric sugars for downstream fermentation. Because glucan and xylan are the major sugars (>90%) in corn stover [1,2], the effectiveness of ATSE pretreatment on the biomass can be principally evaluated by the degree of conversion of glucan and xylan to monomers. Figure 3 shows the conversion of glucan and xylan through 48 h enzymatic hydrolysis of corn stover after ATSE pretreatments under different conditions. Without any pretreatment, only about 17.5% of glucan and 5.1% of xylan conversions could be obtained, while the enzymatic digestibility of corn stover was greatly improved after ATSE pretreatment and it was positively correlated with pretreatment intensity, particularly for the increase of NaOH loading. For instance, with heat preservation time of 1 h, the glucan conversion increased from approximately 42% to 83% (Figure 3a), when the NaOH loading increased from 0.04 to 0.06 g/g biomass, and the corresponding xylan conversion improved from about 29% to 89% (Figure 3b). This was because increasing NaOH dosage facilitated the lignin removal (Figure 2c), thus leading to better accessibility of enzymes to carbohydrates. However, the heat preservation time over 3 h just had slight impact on the improvement of enzymatic digestibility, as presented in Figure 3.

The best glucan and xylan conversions could be obtained after ATSE pretreatment with NaOH loading of 0.08 g/g biomass and 10 hours heat preservation, and the comparable results could be achieved with the NaOH loading of 0.06 g/g biomass as well, with less xylan loss as shown in Figure 2b. Samples pretreated with NaOH loading of 0.04 g/g biomass showed poor enzymatic digestibility which might be due to the inefficient delignification (Figure 2c). However, the xylan conversions of the pretreated samples with NaOH loading of 0.1 g/g biomass are somewhat lower than that with NaOH loading of 0.06 and 0.08 g/g biomass when the preservation time is over 1 h. This is possibly because part of carbohydrates in pretreated biomass is more easily digested than others under the same hydrolysis conditions, and the carbohydrates which are more easily digested in hydrolysis are easier to be degraded in pretreatment as well. Hence, the lower xylan conversion of the pretreated samples with NaOH loading of 0.1 g/g biomass is possibly due to the serious degradation of more easily digestible xylans during ATSE pretreatment, and thus the overcharge of NaOH loading (above 0.1 g/g biomass) is not needed for ATSE pretreatment.

### Scanning electron microscopy (SEM)

**Figure 4:**
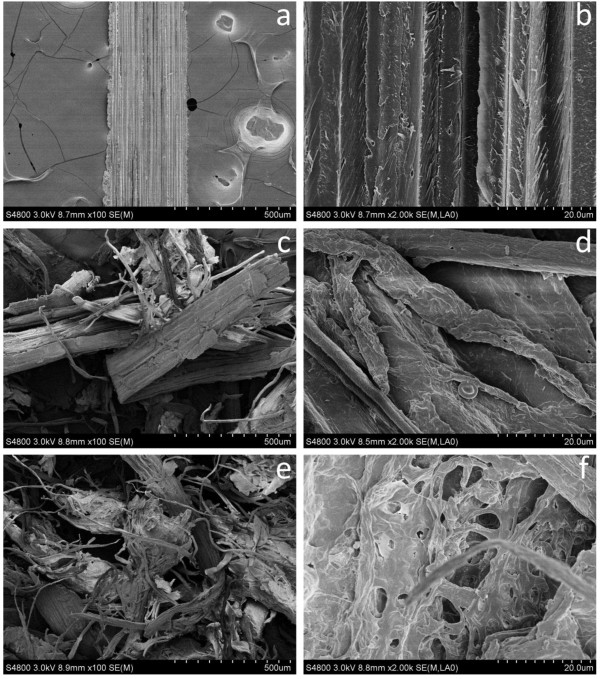
**SEM images of untreated and pretreated corn stover before enzymatic hydrolysis. a**, **b**: raw material; **c**, **d**: NaOH loading of 0.04 g/g biomass and preservation time of 1 h; **e**, **f**: NaOH loading of 0.06 g/g biomass and preservation time of 1 h.

Figure 4 shows SEM images of the untreated and pretreated corn stover samples at different magnifications. It is seen that the raw corn stover has an intact surface structure (Figure 4a and b). However, after pretreatment, the integrated structure of corn stover was broken into separated fibers and fiber bundles, which twisted together (Figure 4c and e). At the same time, the fiber surface became coarse with fiber delamination (Figure 4d and f). This kind of mechanical size reduction can increases external/internal specific surface area, which benefits the enhancement of enzymatic saccharification of biomass [23]. Increasing NaOH loading from 0.04 to 0.06 g/g biomass, more individual fibers were separated with more perforated structure (Figure 4c-f). The increased porosity of corn stover improved the digestibility of biomass as well.

However, the pretreated fibers became irregular with different widths, but they have not been completely separated into single fibers in the conditions used. These structural changes are mainly so called Class I size reduction with a small quantity of Class II size reduction [7]. More importantly, the twin extruder can acts as an effective mixer to accelerate the chemical penetration besides physical breaking.

### Sugar yield and mass balance of ATSE pretreatment

A good enzymatic digestibility of substrate is not the sole goal of pretreatment. Recovering available sugars as much as possible from biomass is also critical for an ideal pretreatment process. The solid and sugar yields are shown in Table 1. Due to the poor hydrolysis conversion, the total sugar yields are only from 26% to 40% at NaOH loading of 0.04 g/g biomass. Increasing NaOH loading from 0.04 to 0.06, 0.08 and 0.1 g/g biomass provided much better yields, while the sugar yield seemed to be slightly influenced by preservation time in most cases. However, heat preservation step is needed. After 1 hour heat preservation the total sugar yields increased by 4%-25% at the different NaOH loading, compared to 0 hour heat preservation. It is undoubtedly that a higher chemical charge will cause degradation of polysaccharides. Therefore, increasing the NaOH loading might not always achieve a higher sugar yield. For example, increasing the NaOH loading from 0.06 to 0.08 or 0.1 g/g biomass at preservation time of 10 h resulted in a 2%-9% reduction of total sugar yield. This is in agreement with the xylan recovery shown in Figure 2b. Moreover, because the susceptibility of xylan at strongly alkaline conditions caused partial loss of xylan into the spent liquor, the glucose yields are higher than xylose yields at all conditions.

**Table 1:** Solid and sugar yields of pretreated corn stover at different pretreatment conditions

**Pretreatment conditions**			**Sugar yield**^**b **^**(%)**		
**NaOH loading (g/g biomass)**	**Preservation time (hour)**	**Solid yield**^**a**^	**Glucose**	**Xylose**	**Total sugar**
0.04	0	77.3	33.6	13.3	26.4
	1	73.8	41.7	22.8	35.0
	2	72.2	42.1	22.0	35.0
	3	72.1	41.7	25.8	36.1
	10	70.0	41.2	36.2	39.5
0.06	0	70.2	57.3	45.5	53.1
	1	65.8	82.0	69.4	77.6
	2	63.0	79.8	68.8	75.9
	3	62.8	85.1	69.7	79.7
	10	62.2	86.2	69.2	80.2
0.08	0	61.7	75.4	58.0	69.3
	1	59.2	85.0	63.8	77.5
	2	57.8	84.7	65.7	78.0
	3	57.4	85.5	62.9	77.5
	10	57.0	86.6	63.4	78.4
0.1	0	54.8	79.6	49.7	69.1
	1	53.5	85.5	51.4	73.4
	2	53.1	83.4	50.8	71.9
	3	52.5	83.1	49.6	71.3
		10	52.2	84.7	47.1	71.4

^a^ solid yield = (Pretreated biomass (g)/Original biomass (g)) × 100; ^b^ glucose yields% = M _glucose in enzymatic hydrolyzate_ × 0.9/M _glucan in original biomass_, xylose yields% = M _xylose in enzymatic hydrolyzate_ × 0.88/M _xylan in original biomass_, total sugar yield% = (M _glucose in enzymatic hydrolyzate_ × 0.9+ M _xylose in enzymatic hydrolyzate_ × 0.88)/( M _glucan in original biomass_ + M _xylan in original biomass_). M is the mass of sugar (g). Data represented are the averages of the results from duplicated experiments.

The maximum yield of total sugar (80.2%) was detected at NaOH loading of 0.06 g/g biomass and 10 h preservation time. With NaOH loading of 0.06 g/g biomass and 1 h preservation time, a similar yield of 77.6% could be obtained. Considering extending preservation time from 1 to 10 h would add much cost to the overall process (e.g., more preservation tanks or much bigger continuous conveyor), 1 hour could be the suitable heat preservation with slightly lower sugar conversion. Therefore, based on the results stated above, NaOH loading of 0.06 g/g biomass with the subsequent heat preservation of 1 h was thought to be the appropriate conditions for ATSE pretreatment. Under such conditions, about 90% of the original polysaccharides were conserved in the pretreated solid (glucan and xylan) and 71% lignin reduction could be achieved after the ATSE pretreatment.

In addition, the effect of different screw speed on pretreatment has been tested. The result indicated that the screw speed had minor effect on the experimental results (Changing the screw speed from 100 to 325 rpm only caused 1-3% variation of total sugar yield. The detailed data was not shown in this paper). In this study the screw speed was fixed at 325 rpm. Meanwhile, the corn stover was fed continuously at a rate of 200 kg/h. The corresponding extrusion duration and energy consumption are about 30 seconds and 240 kWh/ton biomass, respectively.

**Figure 5:**
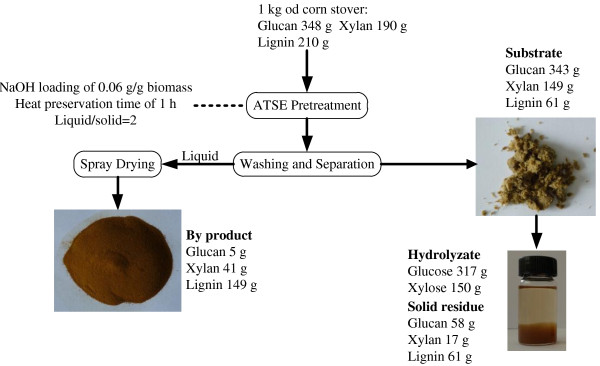
Mass balance of ATSE pretreatment.

An overall mass balance diagram is presented in Figure 5, which is based on the pilot tests of ATSE pretreatment, as well as the lab tests of the spray-drying of spent liquor and enzymatic hydrolysis. The mass balance was performed on the biomass pretreated at the conditions of NaOH loading of 0.06 g/g biomass and preservation time of 1 h. The insoluble fraction corresponding to 66% of the original material was washed and separated from the pretreated slurry prior to enzymatic saccharification. The lignin was recovered with a spray dryer (FH-1500, Shanghai Gaoji, China) without any purification. The *M*_w_, *M*_n_, and PDI (polydispersity index) of the recovered lignin are 22780, 16430, and 1.386, respectively, and the measurement procedure of molecular weight was reported previously [24]. Also, the recovered lignin with relatively larger molecular and small PDI could be modified to value-added products, such as concrete additives and phenol-formaldehyde resins [25]. Finally, the mass balance demonstrates that the total sugar yield is 467 g/kg original biomass which is corresponding to about 78% potential carbohydrates in raw biomass.

### Outcomes and limitations of this research

In the past few years, many types of extrusion pretreatments have been tested, as listed in Table 2. These data is gained from different feedstock and labs. It’s hard to say which one is better. But comparing their experimental conditions and pretreatment types will be still helpful to understand the distinguishing characteristics between these methods and ATSE. Extrusion pretreatments without adding chemicals are environment friendly, but their relative low sugar yield cannot meet the requirement of pretreatment process and the high temperature might increase the process cost [15,26-28]. With the supplement of chemicals like NaOH [16,22,29,30], extrusion pretreatment could yield relatively good total sugar yields (about 82%-88%), but their treatment temperatures were higher than 100°C (114-220°C). Thus, extra heating device is needed, and this will increase the equipment investment and operating cost. Acid can also be used in extrusion treatment [31], but formation of fermentation inhibitors and unavoidable equipment corrosion limit its application. In addition, as reported in these literatures, a size-reduction was needed prior to the extrusion (the raw biomass was ground into 1.4-8 mm), while the biomass just needed to be cut into 2-5 cm pieces as the convenient feedstock before the ATSE pretreatment. Except Kadam KL, Chin CY and Brown LW [16], other extrusion treatments were conducted using lab-scale facilities and their reproducibility and feasibility of pretreatment via larger scale equipment need to be further studied.

**Table 2:** The comparison of extrusion methods

**References**	**Feedstock/size**	**Pretreatment methods**	**Optimum conditions**	**Sugar yields (%)**		
				**Glucose**	**Xylose**	**Total**
Kadam KL, Chin CY and Brown LW [16]	Corn stover/through 1/2” screen	A pilot-scale twin-screw extruder; autohydrolysis followed by NaOH treatment	Autohydrolysis at 210°C followed by NaOH (0.06 g/g biomass) at 220°C, 6 liquid/1 biomass, extruder speed of 28 rpm	80^a^	60	-
Karunanithy C and Muthukumarappan K [15]	Corn stover/4 mm	Single screw extruder; no chemical applied	125°C, extruder speed of 75 rpm, 21% moisture content	75	49	61
Karunanithy C and Muthukumarappan K [29]	Prairie cord grass/8 mm	Alkali soaking followed by single screw extrusion	114°C, extruder speed of 122 rpm, 1.7% NaOH concentration, 7 liquid/1 biomass	86.8	84.5	82.0
Karunanithy C and Muthukumarappan K [30]	Switchgrass/6 mm	Alkali soaking followed by single screw extrusion	180°C, extruder speed of 118 rpm,2% NaOH concentration, 7 liquid/1 biomass	90.5	81.5	88.0
Karunanithy C and Muthukumarappan K [26]	Swithgrass/8 mm	Single screw extrusion; no chemical applied	176°C, extruder speed of 155 rpm, moisture content 20%	41.4	62.2	47.4
Zhang S, Keshwani DR, Xu Y and Hanna MA [22]	Corn stover/2 mm	Alkali soakage followed by twin screw extrusion	140°C, screw speed of 80 rpm, NaOH loading of 0.04 g/g biomass, 3.67 g/min biomass	86.8	50.5	-
Zhang S, Xu Y and Hanna MA [27]	Corn stover/2 mm	Twin-screw extruder; no chemical applied	140°C, screw speed of 80 rpm, 27.5% moisture content, 3.67 g/min biomass	49	25	40
Karunanithy C, Muthukumarappan K and Gibbons WR [28]	Pine wood/8 mm	Single screw extruder; no chemical applied	180°C, screw speed of 150 rpm, 25% moisture content	65.8	65.6	66.1
Choi CH and Oh KK [31]	Rape straw/1.40-2.36 mm	Twin screw extrusion with sulfuric acid	165°C, screw speed of 19.7 rpm, 3.5% sulfuric acid concentration, 6.9 mL/min liquid,0.5 g/min biomass	70.9^b^	-	-
ATSE method (present work)	Corn stover/2-5 cm	Twin screw extrusion with NaOH solution	99°C, screw speed of 325 rpm, NaOH loading of 0.06 g/g biomass, 2 liquid/1 biomass, 200 kg/h biomass	82	69	78

^a^ sugar yield based on the carbohydrate in the raw material; ^b^ glucan digestibility based on the carbohydrate in the pretreated solid. Some details and data of the experiment were not listed in the literatures and not listed equally in this table.

Overall, comparing with other extrusion pretreatments (Table 2), the ATSE pretreatment method exhibits several desirable characteristics: high sugar yield, no need of severe size-reduction prior to the extrusion, relatively low temperature (99°C) and no extra heating needed. In addition, ATSE can use the mature technologies (e.g. chemical recovery system and waste water treatment system) which have been well developed in the modern pulp industry, and the ATSE pretreatment system can be integrated into the alkali-based pulp mill to further lower the capital cost. Thus, there will be few technological barriers and risks to carry out ATSE pretreatment system (Figure 1) at commercial scale.

Elander et al. [32] investigated comparative sugar yield data on the conversion of corn stover to sugars by several leading pretreatment technologies including dilute sulfuric acid, hot water, ammonia fiber expansion, ammonia recycle percolation, sulfur dioxide steam explosion, lime. Comparing ATSE with these leading pretreatment processes is also important. The acidic and non-catalyzed pretreatment operate at relatively high temperatures (130-210°C) and pressures. The high pressurized reactors and corrosive spent liquor required high capital investment. AFEX can be referred as a “dry” process as the chemical can effectively penetrates biomass and only small amounts of steam are needed to achieve the relatively low temperatures. But similar kind of alkaline pretreatments (such as ammonia recycled percolation) often requires a complex chemical recovery system or large amounts of catalysts. The reaction times of the various pretreatments range from a few minutes to 30 min except for lime pretreatment, where residence time ranges from a few hours to several weeks. Moreover, the reported total sugar yields (85-95%) of these processes are higher than that of ATSE process. Comparing ATSE with these different kinds of pretreatment performed in different labs is complicated and it’s hard to draw a conclusion at present situation. A further study using identical materials, analytical methods and enzymatic hydrolysis assays is needed [32]. However, ATSE method also has some possible defects. The chemical recovery processes contribute substantially to the economy of pulp manufacture [33,34], although a modern pulp mill can, as a matter of fact, be self-sufficient in steam and electrical power [33]. Corn stover like other non-wood materials such as wheat straw, reed and bamboo has high content of silicon [35-37], which will react with sodium hydroxide in the pretreatment process, forming sodium silicate, and dissolving in spent liquor [38]. The silicate might cause serious problems in the processes of evaporation and causticizing during the chemical recovery [38,39]. Sodium carbonate pretreatment can improve the enzymatic saccharification of rice straw and keep most of silica in pretreated solid [40], and the causticizing step is not needed during recovering sodium carbonate. Thus, sodium carbonate might be a good substitute of sodium hydroxide during ATSE pretreatment. Moreover, the extrusion machine generated massive friction which might cause mechanical wear and therefore increased the equipment cost. The ATSE can be conducted at a relative low temperature without extra heating, but electric power is needed to crush the biomass. Whether the ATSE is more energy-efficient is still a question in comparison with traditional methods. Thus, the economic feasibility of ATSE need to be further studied.

## Conclusions

This study showed that the ATSE pretreatment effectively disrupted the lignin-carbohydrate complex and greatly improved the enzymatic digestibility of corn stover. A delignification of 71% and total sugar yield of 78% could be achieved with NaOH loading of 0.06 g/g biomass and solid-to-liquid ratio of 1:2 at 99°C. Thus, the ATSE pretreatment can be considered as a promising approach to achieve the efficient conversion of lignocellulosic biomass to fermentable sugars. However, this process is still in the infant stage, and more efforts are needed to understand the mechanism, optimize the operation and further decrease process costs.

## Methods

### Materials

Corn stover used in this study was collected in Qingdao, Shandong Province, China. Before pretreatment, the corn stover was cut into 2-5 cm pieces in length. The chemical composition of the corn stover (on dry weight basis) was listed in Table 3. All experiments were performed in duplicate under the same conditions.

**Table 3:** Chemical composition of corn stover

**Component**	**Content (%)**
Glucan	34.8
Xylan	19.0
Arabinan	1.9
Galactan	1.7
Acid-insoluble lignin	21.0
Ash	2.4
Extractives^a^	17.4

^a^ water soluble and ethanol soluble material.

Commercial enzymes, Celluclast 1.5 L (cellulase) and Novozyme 188 (β-glucosidase), were used as received from Sigma-Aldrich. Sodium hydroxide, sulfuric acid and other chemicals were obtained from local suppliers. All chemicals were of analytical quality and used without any purification.

### ATSE pretreatment

**Figure 6:**
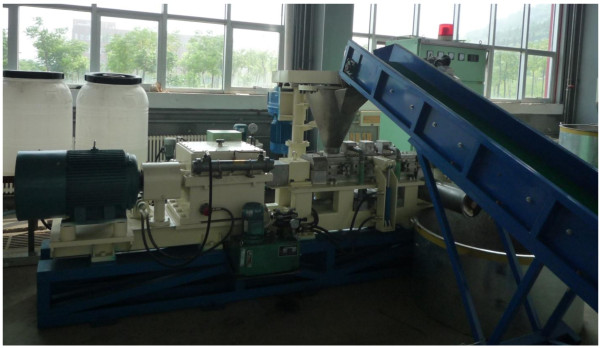
A photo of the pilot-scale extruder.

The ATSE experiments were conducted according to the schematic diagram shown in Figure 1, and two major factors of NaOH dosage and heat preservation time were investigated. The alkali to biomass ratio ranged from 0.04 to 0.1 g NaOH/g oven dried corn stover. The corn stover was pretreated with heat preservation time of 0, 1, 2, 3 and 10 h (Heat preservation time of 0 h represents that after coming out of the extruder the biomass was washed in three minutes and then analyzed without further heat preservation step). A pilot-scale extruder, manufactured by Tianzheng Co. in China, was used with the capacity of 200 kg oven dry corn stover per hour and the picture of the equipment is showed in Figure 6. The corn stover entered the extruder via a feed inlet continuously and was crushed by a twin-screw extruder. Firstly, small amount of water and most of the air in the biomass was removed for improving subsequent penetration of alkali liquor. Then NaOH solution was pumped into the machine with a biomass/liquid ratio of 1/2 (w/w). The high solid/liquid ratio had an advantage on the waste water treatment and energy conservation. A higher biomass/liquid ratio than 1/2 is unavailable in present equipment. Out of the extruder, the sample was sealed in plastic bags and preserved at ambient temperature without any heating for specified time (heat preservation time). In the real factory, the hot pretreated biomass can be carried by a continuous insulated conveyor, in which the preservation step can be accomplished, to maintain the continuity of production. After the preservation, the sample was washed with tap water through a 300 mesh nylon cloth until neutrality. After the washing, the sample was kept in a fridge at 4°C for further experiments.

### Mechanism of the twin-screw extrusion

**Figure 7:**
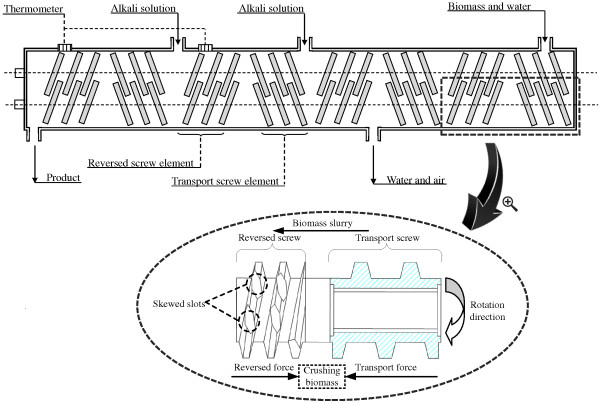
Schematic diagram of the specially designed twin-screw extruder.

The ATSE pretreatment is based on the use of a co rotating twin-screw extruder as main treatment device. Figure 7 shows the basic operating principle of twin-screw extruder, which consists of two co rotating intermeshing screws inside. Each screw consists of four transport screw elements (TSE) and four reversed screw elements (RSE). The biomass enters the main body of extruder via a feed inlet and then is pushed to the RSE by the action of TSE. RSE is the main screw element with threads whose pitch is opposite to the TSE. This results in accumulation and compression of the biomass fiber in the space between TSE and RSE as shown in Figure 7. After crushed by transport force and the reversed force, generated by TSE and RSE respectively, the biomass is forced to pass through the skewed slots of the RSE. Then the biomass enters the next section of TSE and RSE to be further squeezed and crushed. As repeated in this way, the biomass continuously passes through the twin-screw and thus is mechanically crushed.

During the extrusion process, great mechanical forces (frictional force and shearing force) are generated among the biomass fibers and the screws. The high mechanical forces cause fibrillation and shortening of biomass fibers. In addition, during the extrusion process in ATSE machine, tremendous mechanical forces lead to the generation of a great amount of heat thereby significantly increasing the temperature of biomass. In particular, the heat generated by mechanical forces can elevate the temperature to about 99°C (measured accurately by thermometers inside the ATSE machine), so that without additional heat, the pumped alkali can be thoroughly mixed and reacted with the crushed biomass under proper conditions.

### Composition analysis

After washing, the ATSE pretreated solids were dried in an oven at 45°C to constant weight and comminuted using a blender. The composition (including moisture, glucan, xylan, lignin, ash content, etc.) of the pretreated and untreated corn stover was analyzed following the modified National Renewable Energy Laboratory (NREL) Analytical Procedure [41]. The cellulose and hemicellulose content of biomass was determined based on monomer content measured after a two-step acid hydrolysis. The first step with 72% (w/w) H_2_SO_4_ at 25°C for 120 min was used. In the second step, the reaction mixture was diluted to 4% (w/w) H_2_SO_4_ and autoclaved at 120°C for 1 h. After that, sugar content of the hydrolysis liquid was determined by high performance liquid chromatography (HPLC) in an Agilent 1200 system equipped with a refractive index detector. Sugar Pak I guard and analytical columns were used at 80°C with ultrapure water as a mobile phase (0.5 mL/min). The acid insoluble lignin (AIL) presented in the acid hydrolysis residue was determined gravimetrically, and the lignin reduction was expressed as a percent loss of AIL on the basis of the amount of AIL in raw corn stover. The glucan and xylan recovery were presented as percentage of glucan and xylan content in the pretreated corn stover divided by the initial glucan and xylan content in the raw materials. All of the analytical determinations were performed in duplicate. Relative standard deviations were below 5% in all cases.

### Enzymatic hydrolysis

The pretreated solid materials were enzymatically hydrolyzed using NREL standard [42] to assess the effectiveness of pretreatment at different conditions.

Briefly, the experiments were carried out with 2% of substrate solid (w/v) at 50°C in a shaking incubator (Nuoji instruments CO., Model SHZ-A, Jintan, China) at 100 rpm. A mixture of Celluclast 1.5 L with an activity loading of approximately 20 FPU (filter paper unit)/g substrate and Novozyme 188 with an activity loading of about 5 IU (international unit)/g substrate was added together with 20 mL sodium citrate buffer (pH 4.8) for each enzymatic hydrolysis test. The activities of cellulase and β-glucosidase were 121 FPU/mL and 426 IU/mL, respectively, as determined using the methods reported by Ghose [43]. To each vial, 200 μL of a 2% sodium azide solution was added to prevent the growth of organisms during the digestion. Finally, the supernatant was filtered through a 0.22 μm membrane and stored at -4°C for further analysis. The hydrolysate was analyzed with the HPLC system to determine the released sugars. The hydrolysis performance of glucan and xylan in the biomass were evaluated as follows:

$$\begin{array}{l} \mathrm{Glucan}\;\mathrm{conversion}\;\left(\%\right)=M_{\mathrm{glucose}\;\mathrm{in}\;\mathrm{hydrolyzate}}\\ \;\times 0.9/M_{\mathrm{glucan}\;\mathrm{in}\;\mathrm{pretreated}\;\mathrm{biomass}} \end{array}$$

$$\begin{array}{l} \mathrm{Xylan}\;\mathrm{conversion}\;\left(\%\right)=M_{\mathrm{xylose}\;\mathrm{in}\;\mathrm{hydrolyzate}}\\ \;\times 0.88/M_{\mathrm{xylan}\;\mathrm{in}\;\mathrm{pretreated}\;\mathrm{biomass}} \end{array}$$

Wherein, M is the mass of carbohydrate (g).

0.9 is the conversion factor of glucose to equivalent glucan, while 0.88 is the conversion factor of xylose to equivalent xylan. Data presented in this paper are the averages of the results from duplicated experiments.

### SEM

Imaging by SEM was performed using a Hitachi cold field SEM S-4800. After air drying, the samples were mounted on specimen stubs using carbon tape and coated with platinum. Imaging was performed at a beam with the accelerating voltages from 3 kV.

## Abbreviations

ATSE: Alkaline twin-screw extrusion; AFEX: Ammonia fiber explosion; SEM: Scanning electron microscopy; Mw: Weight-average molecular weight; Mn: Number-average molecular weight; PDI: Polydispersity index; TSE: Transport screw element; RSE: Reversed screw element; NREL: National Renewable Energy Laboratory; HPLC: High performance liquid chromatography; AIL: Acid insoluble lignin; FPU: Filter paper unit; IU: International unit; M: Mass of carbohydrate/lignin, g.

## Competing interests

The process described in this paper has been included in a patent application.

## Authors’ contributions

CL completed major experiments and analyzed the data. BL, GY and HW operated the pilot-scale pretreatment equipment and participated in the pretreatment experiments. CL, EH and BL wrote the manuscript. HW and XM designed the project, supervised the experiments and finalized the manuscript. All authors read and approved the final manuscript.
